# Multi-center matched cohort study of convalescent plasma for hospitalized patients with COVID-19

**DOI:** 10.1371/journal.pone.0273223

**Published:** 2022-08-18

**Authors:** Cindy Ke Zhou, Monica M. Bennett, Carlos H. Villa, Kendall P. Hammonds, Yun Lu, Jason Ettlinger, Elisa L. Priest, Robert L. Gottlieb, Steven Davis, Edward Mays, Tainya C. Clarke, Azadeh Shoaibi, Hui-Lee Wong, Steven A. Anderson, Ronan J. Kelly

**Affiliations:** 1 Office of Biostatistics and Epidemiology, Center for Biologics Evaluation and Research, Food and Drug Administration, Silver Spring, Maryland, United States of America; 2 Baylor Scott & White Research Institute, Dallas, TX, United States of America; 3 Office of Blood Research and Review, Center for Biologics Evaluation and Research, Food and Drug Administration, Silver Spring, Maryland, United States of America; 4 Baylor University Medical Center, Dallas, Texas, United States of America; 5 Baylor Heart and Vascular Hospital, Dallas, Texas, United States of America; 6 Baylor Scott and White The Heart Hospital, Plano, Texas, United States of America; 7 Texas A&M Health Science Center, Dallas, Texas, United States of America; 8 TCU and University of North Texas Health Science Center, Fort Worth, Texas, United States of America; 9 Baylor Scott & White Medical Center–Irving, Irving, Texas, United States of America; 10 Center for Biologics Evaluation and Research, Food and Drug Administration, Silver Spring, Maryland, United States of America; 11 Charles A. Sammons Cancer Center Baylor University Medical Center, Dallas, Texas, United States of America; University Campus Bio-Medico di Roma, ITALY

## Abstract

**Background:**

Although frequently used in the early pandemic, data on the effectiveness of COVID-19 convalescent plasma (CCP) remain mixed. We investigated the effectiveness and safety of CCP in hospitalized COVID-19 patients in real-world practices during the first two waves of the pandemic in a multi-hospital healthcare system in Texas.

**Methods and findings:**

Among 11,322 hospitalized patients with confirmed COVID-19 infection from July 1, 2020 to April 15, 2021, we included patients who received CCP and matched them with those who did not receive CCP within ±2 days of the transfusion date across sites within strata of sex, age groups, days and use of dexamethasone from hospital admission to the match date, and oxygen requirements 4–12 hours prior to the match date. Cox proportional hazards model estimated hazard ratios (HRs) and 95% confidence intervals (CIs) for effectiveness outcomes in a propensity score 1:1 matched cohort. Pre-defined safety outcomes were described. We included 1,245 patients each in the CCP treated and untreated groups. Oxygen support was required by 93% of patients at the baseline. The pre-defined primary effectiveness outcome of 28-day in-hospital all-cause mortality (HR = 0.85; 95%CI: 0.66,1.10) were similar between treatment groups. Sensitivity and stratified analyses found similar null results. CCP-treated patients were less likely to be discharged alive (HR = 0.82; 95%CI: 0.74, 0.91), and more likely to receive mechanical ventilation (HR = 1.48; 95%CI: 1.12, 1.96). Safety outcomes were rare and similar between treatment groups.

**Conclusion:**

The findings in this large, matched cohort of patients hospitalized with COVID-19 and mostly requiring oxygen support at the time of treatment, do not support a clinical benefit in 28-day in-hospital all-cause mortality for CCP. Future studies should assess the potential benefits with specifically high-titer units in perhaps certain subgroups of patients (e.g. those with early disease or immunocompromised).

## Introduction

Transfusion of convalescent plasma (CP) is frequently proposed as passive immune therapy for emerging viral infections, including during the coronavirus disease 2019 (COVID-19) pandemic caused by SARS-CoV-2 [1]. The rationale is that antibodies can be transferred from recovered patients and theoretically treat or prevent disease [2]. While CP is one of the earliest available therapies for emerging infectious diseases, data on its effectiveness have historically been limited by non-randomized designs [3]. Based on available data, limited alternative treatment options, and historical precedents, the U.S. Food and Drug Administration (FDA) determined in March 2020, that COVID-19 convalescent plasma (CCP) was eligible for use under expanded access, as well as in controlled trials, under an active Investigational New Drug application (IND). Initially, CCP was provided under individual patient emergency IND expanded access, followed by a national expanded access protocol (EAP) sponsored by the Mayo Clinic [4], and subsequently under terms of an Emergency Use Authorization (EUA) in August 2020. In February 2021, based on updated findings of randomized controlled trials (RCTs), the EUA was restricted to the use of CCP with high titers of anti-SARS-CoV-2 antibodies early in the course of disease or in patients with impaired humoral immunity. In December 2021, with emerging evidence from RCTs and observational studies of CCP, the EUA was further restricted to the use of high titers plasma for the treatment of COVID-19 in patients with immunosuppressive disease or receiving immunosuppressive treatment [5].

While RCTs provide more definitive evidence of treatment efficacy, analyses of the real-world use of CCP in large cohorts may offer insight into clinical effectiveness, and reasons for differences across observational studies, including assessment of concomitant therapies. Identifying factors potentially associated with the clinical benefit, or lack thereof, can inform further analyses of RCT data, assist in future trial designs, and provide valuable information for the use of CP in future emerging infectious diseases. Finally, methods used to control for confounding can inform future assessment of real-world evidence, especially in emerging pandemics. Herein, we report associations between CCP treatment and effectiveness outcomes, and describe safety outcomes following transfusion in a multi-hospital healthcare system in Texas from July 2020 to April 2021.

## Materials and methods

**Fig 1:**
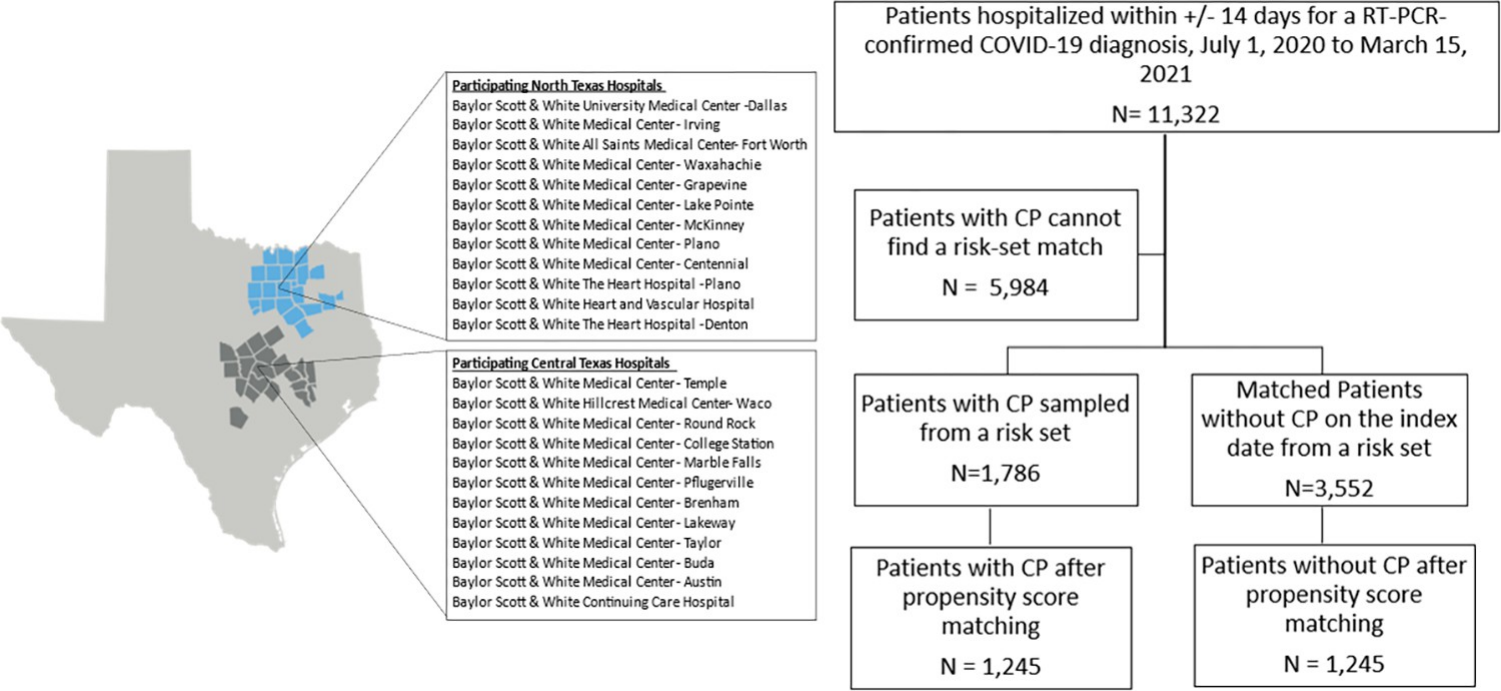
Baylor Scott & White Health participating hospitals and analytic patient identification in this study. Map adapted from Baylor Scott & White Health marketing materials under a CC BY license, with permission from Baylor Scott & White Health, original copyright 2021. Abbreviations: CCP = COVID-19 convalescent plasma.

This retrospective cohort study analyzed data extracted from the Epic electronic health record (EHR) system from 25 acute care Baylor Scott White & Health (BSWH) hospitals across north and central Texas from July 1, 2020 to April 15, 2021 (Fig 1). The protocol was jointly developed by investigators from the FDA and the Baylor Scott & White Research Institute and is published online. This study was reviewed and approved by the Baylor Scott & White Research Institute Institutional Review Board, with a waiver of the requirement for informed consent.

### Analytic cohort

Patients who had a positive RT-PCR test for SARS-CoV-2 infection within 14 days of hospital admission were identified for risk-set sampling. Among these patients, we included patients who received CCP and those who did not receive CCP within four days (± 2 days) of the transfusion date during the study period across sites. Prior to August 23, 2020, patients in the study received CCP under INDs, largely through the national EAP, while patients treated after August 23, 2020, may have received CCP under EUA or IND. Eligibility for treatment under either regulatory pathway included hospitalized patients with COVID-19, and transfused patients in this study were therefore expected to include a heterogenous mix that reflected real-world practice at the time. The heterogeneity of clinical characteristics applied to the untreated group as well. Matching on calendar date was conducted to account for the potential period effect as the standard of care changed quickly for hospitalized COVID-19 patients with the evolving pandemic. The transfusion date or the match day was defined as the index date. We matched each CCP recipient to up to four untreated patients on sex, age (±5 years), days from hospital admission to index date, oxygen requirements 4–12 hours prior to index date, and use of dexamethasone from admission to index date. Specific definitions of oxygen requirement strata (i.e., basic versus advanced oxygenation) are provided in the publicly posted study protocol and generally reflect low versus high flow oxygen requirement (Table 1 in S1 Appendix). We excluded patients who were previously admitted to hospitals due to RT-PCR-confirmed COVID-19 for potentially incomplete capture of COVID-19 treatments, who were discharged on the same day of admission due to lack of follow-up, and whose index date was <28 days from the end of the study period due to inadequate observation for the primary outcome.

### Exposure

COVID-19 patients admitted to a BSWH hospital were considered for CCP transfusion according to disease severity, availability of treatments, and clinician’s judgment. CCP was identified with ISBT-128 [6] code (Table 2 in S1 Appendix) in local Blood Bank data (i.e., SoftBank [SCC] and SafeTrace [Haemonetics]). Depending on blood donor center supplier inventory and availability of testing for titers, including labeling high titer per updated FDA requirement, CCP was sent to the individual hospital blood banks for storage, processing, and thawing per treating physician order. CCP was obtained from three blood donor center suppliers that started antibody testing between October and November 2020. Early on, however, titers were not available for testing. Our preliminary assessment found most CCP-treated patients only received one unit of transfusion. Therefore, the first exposure to CCP was of interest for patients who received multiple units.

### Outcomes

The pre-defined primary effectiveness outcome was 28-day in-hospital all-cause mortality. Secondary effectiveness outcomes included discharge alive, intensive care unit (ICU) transfer, and mechanical ventilation. We identified date of death and discharge status for in-hospital mortality and discharge alive status. ICU transfer was defined as EHR transfer entries in and out of ICU departments, or ICU accommodation codes. Mechanical ventilation was identified by reviewing the oxygen therapy flowsheet for the physician-defined treatment types that met criteria.

Safety outcomes related to plasma transfusion were explored, selected from the Centers for Disease Control and Prevention (CDC) National Healthcare Safety Network (NHSN) Biovigilance Component Hemovigilance Module Surveillance Protocol, and identified with International Classification of Diseases, 10th Revision, Clinical Modification (ICD-10-CM) (Table 1 in S2 Appendix).

### Statistical analysis

Covariates at baseline, defined as the period prior to and including the index date, were reported as frequencies and proportions for categorical variables, and median and interquartile range (IQR) or mean and standard deviation (SD) for continuous variables, as appropriate.

To account for confounding by baseline covariates, we conducted one-to-one nearest neighbor propensity score matching without replacement using a caliper of 0.25 and exact match on age (±2.5 years), days from hospital admission to the index date, oxygen support 4–12 hours prior to the index date, and use of dexamethasone from admission to the index date between exposure groups. Absolute standardized differences were computed for each baseline covariate to check its distribution balance between exposure groups pre- and post-propensity score matching. We also visually inspected overlaps between density plots of post-propensity score matching (Fig 1 in S2 Appendix).

Crude and adjusted incidence rates for all-cause in-hospital mortality in the first 7, 14, 21, and 28 days from the index date were estimated pre- and post-propensity score matching, respectively. Cumulative incidence curves for in-hospital mortality were depicted for each exposure group and compared using the log-rank test. Cox proportional hazards regression estimated hazard ratios (HRs) and 95% confidence intervals (CIs) using the post-propensity score matched cohort for effectiveness outcomes, with hospital site included as the random effect. Hospital site was not included in the propensity score model to preserve the degree of freedom, given the number of other covariates and the sample size. For the primary effectiveness outcome, patients were followed from the index date until the earliest of death, discharge alive, or day 28 from the index date (or the end of the study period in the sensitivity analysis below). The proportional hazards assumption was evaluated using Schoenfeld residuals test and visual inspection of log-log plots. Given the rarity of the safety outcomes, we only reported frequencies and proportions of these among patients who received CCP in the post-propensity score matched population.

To evaluate the robustness of the effectiveness results, we conducted following sensitivity analyses for all pre-defined effectiveness outcomes: 1) restricting to a subset of patients admitted to the hospital within ±7 days of RT-PCR confirmed COVID-19 diagnosis; 2) following patients up to the end of the study period; and 3) estimating sub-distribution hazard ratios using the Fine‐Gray model to account for the competing risk between in-hospital mortality and discharge alive. To further evaluate the CCP effectiveness, we conducted the following subgroup analyses for the pre-defined primary effectiveness outcome: 1) stratifying mechanical ventilation status prior to or at the index date; 2) restricting to patients receiving remdesivir prior to or at the index date; 3) stratifying the time interval from hospital admission to the index date (<72 versus ≥72 hours); and 4) stratifying oxygen support 4–12 hours prior to the index date.

Secondary outcomes (incident of ICU transfer, mechanical ventilation, and discharged alive) were also analyzed to evaluate CCP’s effectiveness on disease progression. Analysis of these outcomes was performed in the same manner as the primary outcome.

All statistical analyses were conducted with SAS 9.4 and R 3.5.1. A two-sided *P*-value of <0.05 was considered statistically significant. Missing values were coded as a separate category for each covariate and included in the analyses. No adjustments were made for multiple testing.

## Results

Fig 1 presents the participating hospital sites and the identification of the analytic cohort. There were 11,322 eligible hospitalized patients testing positive for SARS-CoV-2 within 14 days of admission during the study period, of whom 2,495 received CCP. Risk-set sampling identified 1,786 patients treated with CCP and 3,552 matched patients who did not receive CCP. The propensity score matching resulted in 1,245 patients each in the CCP treated and untreated groups.

### Baseline patient clinical characteristics

Table 1, and Table 2 in S2 Appendix, present the distribution of patient demographics, baseline comorbidities, concurrent medications, proxies for COVID-19 severity, and select laboratory results, pre- and post-propensity score matching. After risk-set sampling (or pre-propensity score matching) and propensity score matching, patient demographics and baseline comorbidities are similarly distributed between exposure groups, with the median age of 66 years, over half of the patients being male, the median length of hospital stay of one day prior to the index date and most commonly occurring comorbidities being hypertension (70%).

**Table 1:** Baseline demographics and clinical characteristics of convalescent plasma treated patients and matched untreated patients through risk-set sampling and propensity score matching.

	CCP Treated	Matched CCP Untreated	Absolute Std Diff
	N = 1245	N = 1245	
***Demographics***			
**Age on the index date (years)**			0.008
Mean (SD)	65 (13.5)	65.1 (13.4)	
Median (IQR)	66 (56, 75)	66 (56, 75)	
**Days from hospital admission to the index date**			0
Mean (SD)	1.4 (1.1)	1.4 (1.1)	
Median (IQR)	1 (1, 2)	1 (1, 2)	
**Sex**			0.049
Female	529 (42.5%)	499 (40.1%)	
Male	716 (57.5%)	746 (59.9%)	
**Race/ethnicity**			0.055
Non-Hispanic, White	649 (52.1%)	646 (51.9%)	
Non-Hispanic, Black	186 (14.9%)	192 (15.4%)	
Hispanic	347 (27.9%)	349 (28.0%)	
Non-Hispanic, Others	45 (3.6%)	40 (3.2%)	
Missing/Unknown	18 (1.4%)	18 (1.4%)	
**Hospital Category** *			0.719
>20 ICU beds	688 (55.3%)	877 (70.4%)	
16–20 ICU beds	68 (5.5%)	216 (17.3%)	
11–15 ICU beds	315 (25.3%)	78 (6.3%)	
≤10 ICU beds	174 (14.0%)	74 (5.9%)	
***Comorbidities***			
**History of cancer excluding non-melanoma skin cancer**	86 (6.9%)	85 (6.8%)	0.003
**Cardiovascular conditions**			
Thrombotic or thromboembolic complications	205 (16.5%)	188 (15.1%)	0.038
Stroke	112 (9.0%)	113 (9.1%)	0.003
Myocardial infarction	78 (6.3%)	70 (5.6%)	0.027
Venous thromboembolism, Deep vein thrombosis, Pulmonary embolism	45 (3.6%)	43 (3.5%)	0.009
Hypertension	875 (70.3%)	874 (70.2%)	0.002
Heart failure	256 (20.6%)	268 (21.5%)	0.024
Cardiac arrhythmias	225 (18.1%)	231 (18.6%)	0.013
**Chronic respiratory disease**			
Chronic obstructive pulmonary disease	233 (18.7%)	227 (18.2%)	0.012
Asthma	137 (11.0%)	132 (10.6%)	0.013
Diabetes mellitus	650 (52.2%)	652 (52.4%)	0.003
**Chronic kidney disease**			0.044
1 to 4	257 (20.6%)	260 (20.9%)	
5 or end-stage renal disease	68 (5.5%)	81 (6.5%)	
**Chronic liver disease**	91 (7.3%)	75 (6.0%)	0.052
**History of organ transplantation**	22 (1.8%)	28 (2.2%)	0.034
**HIV/AIDS**	0 (0%)	1 (0.1%)	0.04
**Obesity at hospital admission**			0.031
BMI 30–39.9	508 (40.8%)	506 (40.6%)	
BMI ≥40	172 (13.8%)	169 (13.6%)	
Missing	2 (0.2%)	1 (0.1%)	
**Pregnant from index date to study completion**	4 (0.3%)	4 (0.3%)	0
***Comedications***			
**Remdesivir**	866 (69.6%)	858 (68.9%)	0.014
**Hydroxychloroquine/Chloroquine**	8 (0.6%)	8 (0.6%)	0
**Azithromycin**	689 (55.3%)	708 (56.9%)	0.031
**Glucocorticoid/Steroids**			
Dexamethasone	1233 (99.0%)	1233 (99.0%)	0
Prednisone	77 (6.2%)	88 (7.1%)	0.036
Hydrocortisone	44 (3.5%)	41 (3.3%)	0.013
Anti-platelet agents	479 (38.5%)	477 (38.3%)	0.003
**Tocilizumab**	41 (3.3%)	47 (3.8%)	0.026
**ACE-Inhibitors /Angiotensin Receptor Blockers**	383 (30.8%)	375 (30.1%)	0.014
**Anti-thrombotic drugs**	1116 (89.6%)	1125 (90.4%)	0.024
***Clinical Characteristics***			
**Oxygenation support 4–12 hours prior to the index date**			0
Room air	85 (6.8%)	85 (6.8%)	
Basic oxygen support	839 (67.4%)	839 (67.4%)	
Advanced oxygen support	309 (24.8%)	309 (24.8%)	
Invasive ventilation	12 (1.0%)	12 (1.0%)	
ECMO	0 (0%)	0 (0%)	
**ICU admission prior to index date**	125 (10.0%)	134 (10.8%)	0.024
**Vital signs closest to the index date**			
** Respiratory rates**			0.012
Mean (SD)	20.6 (4.6)	20.5 (4.6)	
Median (IQR)	20 (18, 22)	20 (18, 22)	
** Heart rate**			0.004
Mean (SD)	78.1 (15.0)	78.1 (16.0)	
Median (IQR)	76 (68, 87)	77 (67, 87)	
** Systolic blood pressure**			0.025
Mean (SD)	129.8 (19.7)	129.4 (19.2)	
Median (IQR)	128 (116, 143)	128 (116, 141)	
** Temperature (F)**			0.016
Mean (SD)	98.3 (0.8)	98.3 (0.8)	
Median (IQR)	98.1 (97.8, 98.6)	98.2 (97.8, 98.6)	
**Laboratory results closest to the index date**			
** Creatinine Level (mg/dL)**			0
≤ 1.5	989 (79.4%)	979 (78.6%)	
>1.5	221 (17.8%)	227 (18.2%)	
Missing	35 (2.8%)	39 (3.1%)	
** D-dimer Level (ug/mL FEU)**			0.059
≤ 1	566 (45.5%)	559 (44.9%)	
1–2	264 (21.2%)	265 (21.3%)	
>2	178 (14.3%)	193 (15.5%)	
Missing	237 (19.0%)	228 (18.3%)	
** Cardiac troponin Level—Tnl (98%) or TnT (2%) (ng/mL)**			0.022
≤ 1	864 (69.4%)	854 (68.6%)	
> 1	25 (2.0%)	20 (1.6%)	
Missing	356 (28.6%)	371 (29.8%)	
** Absolute lymphocyte count Level (K/uL)**			0
<1	820 (65.9%)	816 (65.5%)	
≥1	69 (5.5%)	70 (5.6%)	
Missing	356 (28.6%)	359 (28.8%)	
** Ferritin Level (ng/mL)**			0.057
≤400	281 (22.6%)	294 (23.6%)	
>400	634 (50.9%)	640 (51.4%)	
Missing	330 (26.5%)	311 (25.0%)	
** C-reactive protein Level (mg/dL)**			0.094
≤0.5	18 (1.4%)	15 (1.2%)	
>0.5	967 (77.7%)	988 (79.4%)	
Missing	260 (20.9%)	242 (19.4%)	

Abbreviations: ACE = angiotensin converting enzyme; BMI = body mass index; CCP = COVID-19 convalescent plasma; ECMO = extracorporeal membrane oxygenation; FEU = Forty-foot equivalent unit; ICU = intensive care unit; IQR = interquartile range; SD = standard deviation; Std diff = Standardized difference; Tn = Cardiac troponin.

*Hospital category was not included in the propensity score model but included as a random effect in the outcome regression.

Risk-set sampling resulted in an even distribution of dexamethasone treatment in the pre- and post-propensity score matched cohorts with 99% of patients receiving it in the post-propensity score matched cohort. Propensity score matching improved imbalance in some comedications with absolute standardized differences <0.1; 90% of patients received anti-thrombotic drugs, 70% received remdesivir, and over 50% received azithromycin in each exposure group.

Proxies for COVID-19 disease severity before and after propensity score matching are also presented in Table 1. Risk-set sampling and propensity score matching resulted in the same distribution of oxygen support 4–12 hours prior to index date between exposure groups, with 67% patients requiring basic oxygen support and 25% requiring advanced oxygen support. Distribution of ICU admission and vital signs closest to the index date was similar between exposure groups. After propensity score matching, approximately 11% of patients were admitted to the ICU prior to index date in each exposure group. Laboratory results closest to the index date were balanced between exposure groups after propensity score matching.

### Safety outcomes

There was a single transfusion associated circulatory overload (TACO) incident in the CCP-treated group (Table 3 in S2 Appendix). Incidence of thrombotic or thromboembolic complication (11.2% CCP-treated vs. 10.5% untreated), and cardiac arrhythmias (12.9% CCP-treated vs. 13.7% untreated), were similar between exposure groups. No incidence of other prespecified, transfusion-related safety outcomes was observed.

### Primary effectiveness outcomes

**Fig 2:**
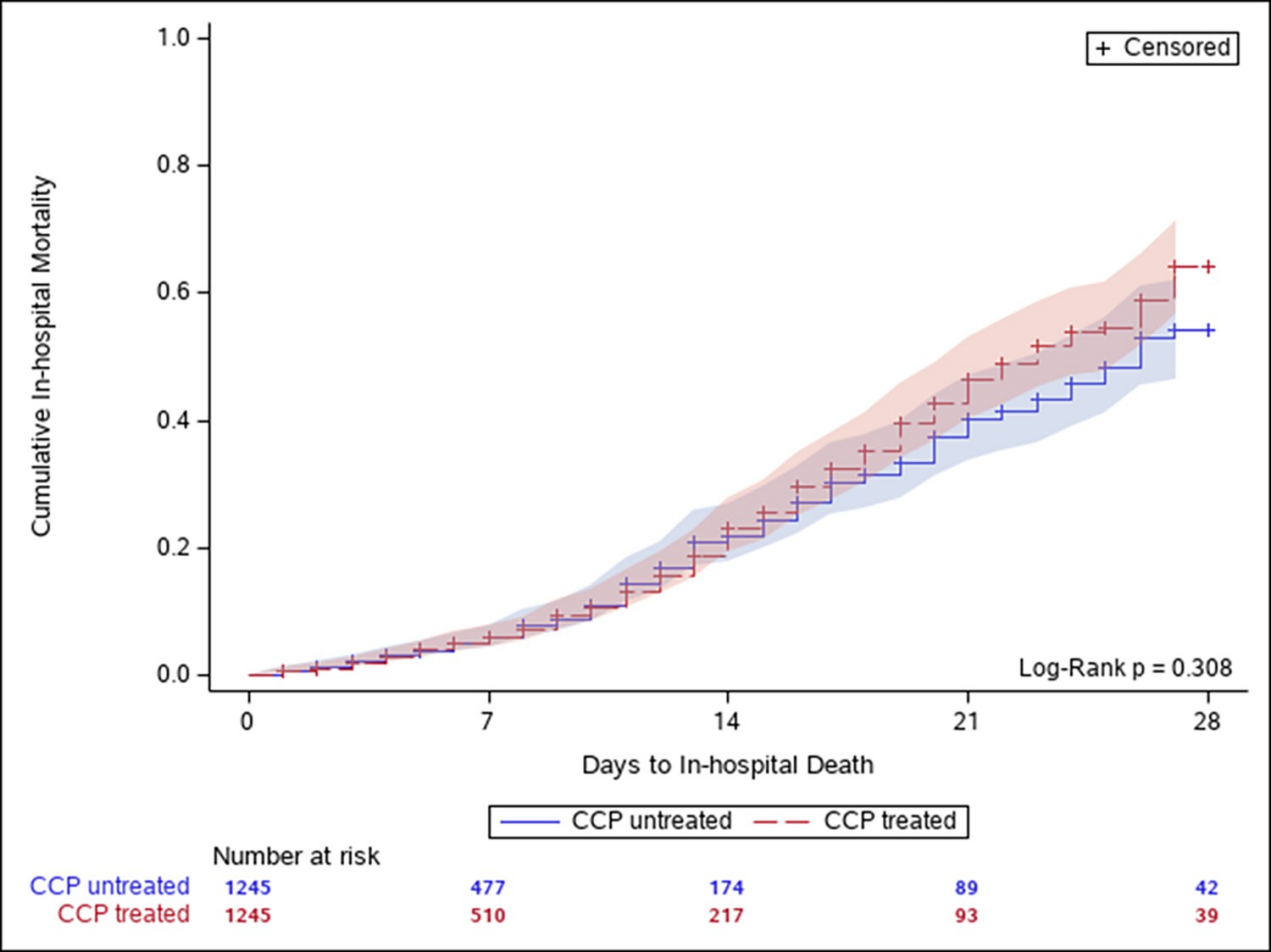
Cumulative in-hospital mortality up to 28 days after the index date. During the study period (July 1, 2020 to April 15, 2021), 179 (14.4%) of 1,245 patients treated with COVID-19 convalescent plasma and 143 (11.5%) of 1,245 1:1 matched untreated patients died in the hospital. The median follow-up time was 5 days (range 1–28) for both the treated and the untreated. Overall, no statistically significant difference was observed for the treated versus the untreated group. Abbreviations: CCP = COVID-19 convalescent plasma.

In-hospital mortality incidence rates (IRs) for both pre- and post-propensity score matched cohorts are given in Table 4 in S2 Appendix. After propensity score matching, there was no statistically significant difference in mortality IRs at follow-up of 7, 14, 21, or 28 days after the index date between exposure groups. Up to 28 days after the index date, the IRs were 18.1 (95%CI: 15.6, 20.9) and 15.7 (95%CI: 13.3, 18.5) for CCP-treated and untreated patients, respectively. Fig 2 demonstrates the overall cumulative incidence of in-hospital mortality was not statistically different in CCP-treated and untreated patients (*P* = 0.308).

**Fig 3:**
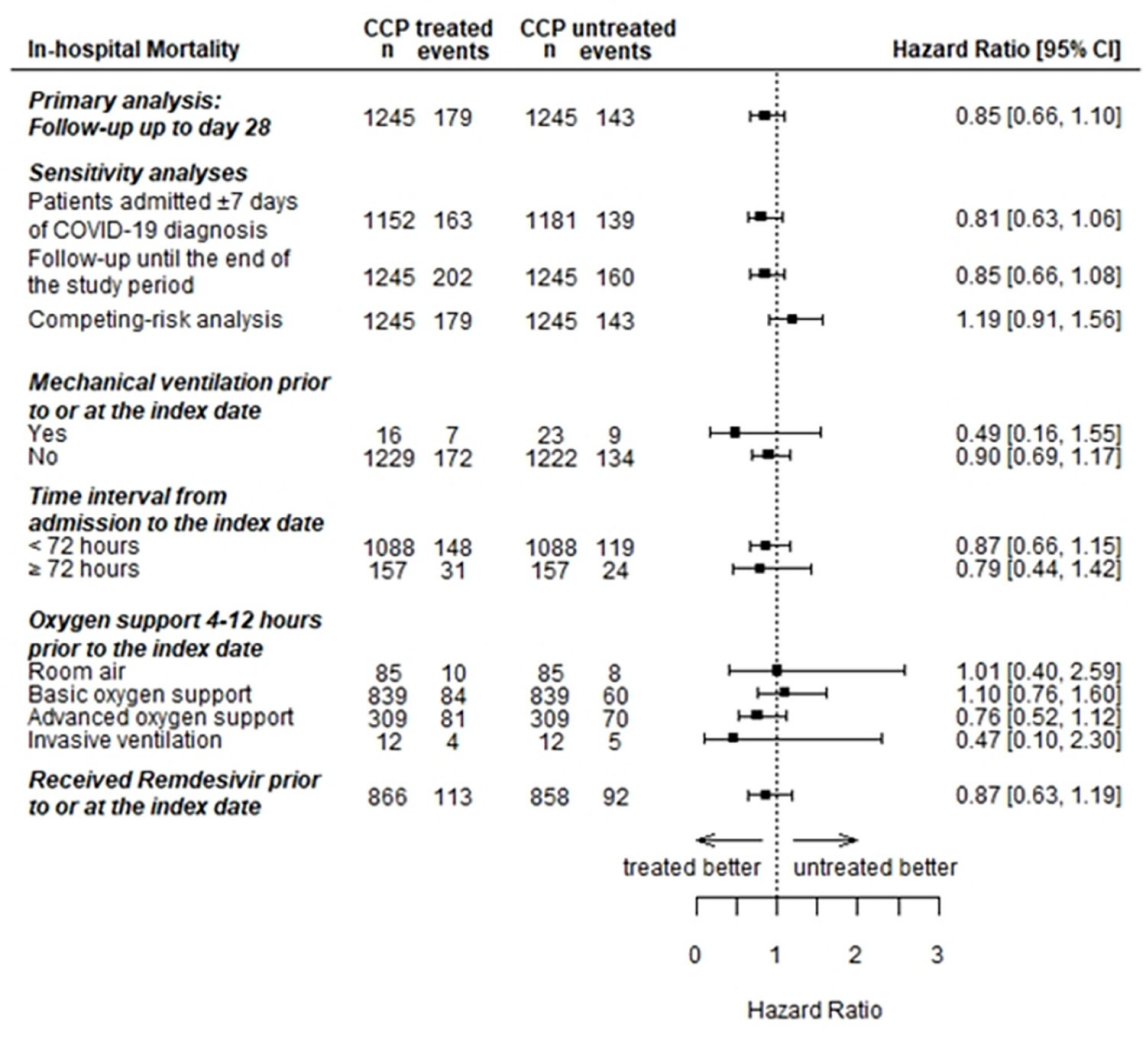
Associations of COVID-19 convalescent plasma (CCP) with 28 day in-hospital mortality in the primary analysis, sensitivity analyses, stratification analyses for mechanical ventilation, time interval from admission to the index date, oxygen support 4–12 hours prior to the index date, and receipt of remdesivir prior to or at the index date. Abbreviations: CCP = COVID-19 convalescent plasma; CI = confidence interval.

Cox proportional hazards modeling found no statistically significant difference for 28-day in-hospital mortality (HR = 0.85; 95%CI: 0.66,1.10) between CCP-treated and untreated patients (Fig 3). Proportional hazards assumption held with null Schoenfeld residuals test (*P* = 0.190) and the interaction term of log(time) and treatment in the Cox model (*P* = 0.295). Similar null findings were also observed in sensitivity analyses, when 1) restricting the analytic cohort to a subset of patients admitted within ±7 days of RT-PCR confirmed COVID-19 (HR = 0.81; 95%CI: 0.63, 1.06); 2) allowing follow-up to continue until the end of the study period (HR = 0.85; 95%CI: 0.66, 1.08); and 3) considering the competing-risk of discharge alive (HR = 1.19; 95%CI: 0.91, 1.56).

Stratification by mechanical ventilation status at the index date found no statistically significant difference for 28-day in-hospital mortality between CCP-treated and untreated patients (Fig 3). Only a small proportion of patients had mechanical ventilation prior to or at the index date (16 CCP-treated, 23 untreated). Similarly, there was no significant difference between exposure groups when stratified by time from admission to index date, or by oxygen support 4–12 hours prior to the index date (Fig 3). Among patients who received remdesivir prior to or at index date, no statistically significant association was observed (HR = 0.87; 95%CI: 0.63, 1.19).

### Secondary effectiveness outcomes

**Fig 4:**
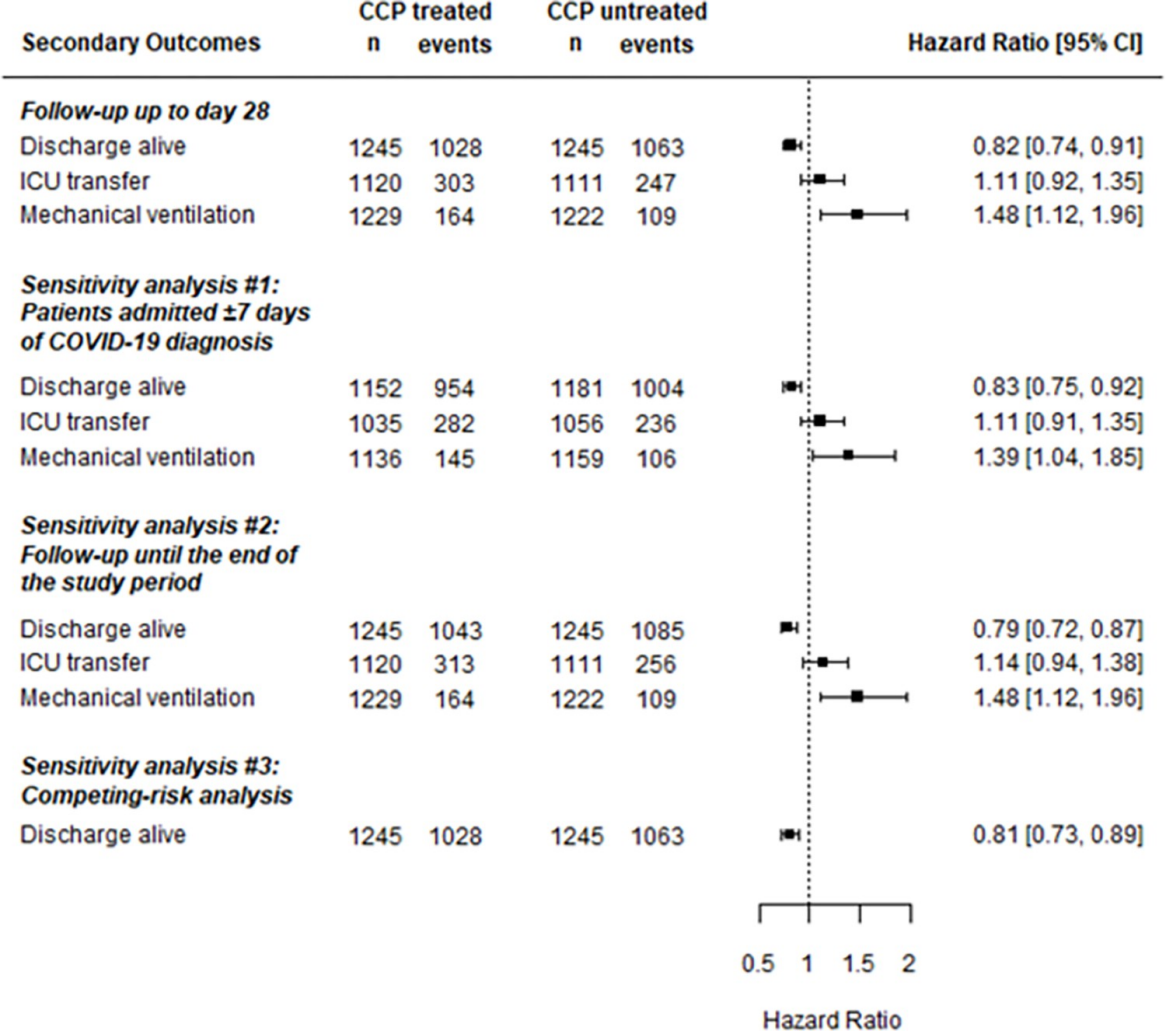
Associations of COVID-19 convalescent plasma (CCP) with secondary effectiveness outcomes in the primary and sensitivity analyses. Abbreviations: CCP = COVID-19 convalescent plasma; CI = confidence interval; ICU = intensive care unit.

Compared with untreated patients, CCP-treated patients were less likely to be discharged alive up to 28 days after index date (HR = 0.82; 95%CI: 0.74, 0.91), and more likely to receive mechanical ventilation (HR = 1.48; 95%CI: 1.12, 1.96) (Fig 4). These results were similarly seen in corresponding sensitivity analyses. However, being treated with CCP was not statistically associated with ICU transfer with follow-up of up to 28 days, or in any of the corresponding sensitivity analyses. A similar discharge disposition between exposure groups was observed (Table 5 in S2 Appendix).

## Discussion

Our study reports results from a large, matched cohort of hospitalized COVID-19 patients and did not find a clinical benefit in in-hospital 28-day all-cause mortality for CCP-treated compared to non-treated patients. Our study assessed the effectiveness of CCP in a population consistent with real-world clinical practices over the first two waves of the pandemic in Texas (i.e., use of CCP of unknown antibody titer across the general hospitalized COVID-19 population, including patients requiring various levels of oxygen support) (Fig 2 in S2 Appendix) and the EAP overall [7]. Crude incidence rates of in-hospital 28-day all-cause mortality in the study population (15.7% and 18.1% in control and treatment arms, respectively) fell between rates seen in RCTs in similar populations, higher than some [8], but lower than a large international RCT [9] and the EAP overall [4]. Further, our study did not find an association of CCP treatment with clinical benefit in subgroups such as those not on mechanical ventilation, those treated within 72 hours of admission, or patients across different levels of oxygen support, although these variables are likely imprecise surrogates of disease severity or immune response. While a statistically significant decreased likelihood of being discharged alive and increased probability of mechanical ventilation (both secondary outcomes) in the CCP-treated group, and a null result for the primary outcome (28-day in-hospital all-cause mortality), all point in the direction of a lack of effectiveness of CCP, the secondary outcomes should be interpreted with caution. These secondary outcomes are based on measures taken in response to physician judgment as would be a decision to transfuse with CCP, and some of the reasons for these clinical decisions can be the potential confounders when examining the effect of CCP, and may not have been accounted for in this study.

Rates of key safety events were similar between exposure groups, and transfusion-associated adverse events were rare, with only one TACO episode reported in the transfused group. Considering the incidence of transfusion-associated adverse events (e.g., TACO and possible transfusion-related acute lung injury) was lower than typically reported for plasma transfusion in hospitalized and critically ill subjects [10–12], these events may be under-captured in EHRs using ICD-10-CM codes only [7].

Evidence of CCP’s role in the treatment of COVID-19 remains mixed. Heterogeneity of clinical practice, clinical characteristics of patient populations, and product characteristics, in addition to the evolving pandemic and concurrent clinical management, complicates the interpretation of meta-analyses [13–15]. Several RCTs of CCP for treatment of moderate-to-severe hospitalized COVID-19 patients, typically at 7 or more days post-symptom-onset, did not find clinical benefits [8, 9, 16–22], although some trials found signals of clinical benefit, sometimes in certain subgroups [21, 23–26]. Assessment of patients treated under the EAP found a modest survival benefit in patients who received high-titer CCP compared to those who received low-titer CCP [27, 28]. A Bayesian reanalysis of RECOVERY argued that it remained plausible that CCP has a small but clinically significant effect on mortality in seronegative patients [29]. In the outpatient setting, some RCTs found a reduction in progression to severe disease [30, 31], while others did not [32], although patient populations, timing of treatment, and product characteristics were again variable. Observational matched cohort studies also found inconsistent results. Some studies found an association with clinical benefit [33–36], which was more likely with high titer CCP used earlier in the course of disease, or in specific subgroups, such as the immunosuppressed [37]. However, other matched cohort studies did not observe clinical benefit in hospitalized patients [38–40]. Small RCTs and observational studies are limited by insufficient statistical power to detect modest but clinically meaningful effects, particularly in subgroups, or by biases due to confounding. While one large observational study described an association between CCP transfusion and improved survival [35], an effect size much larger than expected based on subsequently reported RCT data suggests a potential for residual confounding, indicating a role for additional large observational studies such as the one described herein.

The observed lack of effect in COVID-19 patients in this study should be considered in the context of the anticipated importance of timing of passive immune therapy (such as CCP or monoclonal therapy). The high proportion of subjects already requiring oxygen support (>90%), suggests that many patients would have been more than a week into symptomatic illness [41] and that a large fraction of patients in the current study would have already been seropositive in the absence of impaired immunity [42], although this information was unlikely to be available at the time of treatment. For example, in similar study populations [9, 19, 43], approximately 50% to 70% of those with known serostatus were already seropositive. The emerging consensus signal from studies of inpatient antibody therapies, which deliver high neutralization titers, tempers expectations, as such treatments are likely ineffective if serostatus is not taken into account [44, 45]. A similar pattern was suggested in re-analyses of a large CCP RCT [29]. Clinical factors in recipients may also guide optimal patient selection for CCP therapy or future clinical studies of CCP [46].

Strengths of the current study include a large sample size selected with *a priori* power calculations to detect a relative risk of 0.80. As evidenced by the characteristics summarized in Table 1, our study achieved good comparability between the CCP-treated and untreated groups on measured potential confounders of baseline comorbidities, concomitant therapies, oxygen support requirements, and baseline laboratory findings, all of which may impact the risk of severe COVID-19 and progression to mortality. Finally, the diverse patient characteristics (e.g., race/ethnicity) and clinical practice in a large multi-site healthcare system contributes to the generalizability of the study results. This study contributes to the body of knowledge about the effectiveness of the CCP in COVID-19 hospitalized patients because it is an observational study that is conducted in real-world conditions of the pandemic and not in controlled conditions of an RCT, but the study design and analytic method have been able to mimic the controlled conditions of an RCT to a large extent. The null study results should not diminish its value but be interpreted in the context of a large observational study with a strong study design and analytic method and some limitations.

The current study has notable limitations. First, our study lacks anti-SARS-CoV-2 antibody titer information in the transfused CCP, either via serologic or neutralization testing, which is likely to impact potential effectiveness of the transfused CCP. Nonetheless, as availability of such tests remained limited during the study period (Fig 2 in S2 Appendix) and implementation was delayed in many blood establishments, the use of CCP of unknown titers is expected to have been common during the study period, and therefore this aspect of the study reflects real-world use in many cases. In February 2021, FDA found that low titer CCP no longer met the evidentiary standard for EUA, and the EUA for CCP was revised to restrict use to high-titer CCP early in the course of illness. Second, patients’ baseline antibody titers or other measures of humoral immunity between the treated and untreated patients, which may affect mortality risk and likelihood of benefit for a passive immune therapy, were not assessable. Third, while the exposure groups were well balanced for measured potential confounders, the degree to which unmeasured and residual confounding impacts the findings is uncertain. Finally, the study population of hospitalized COVID-19 patients was heterogeneous with respect to disease severity and largely found to already require various levels of oxygen support and concurrent treatment with systemic steroids at the time of CCP treatment, limiting the power to examine patients earlier in the course of disease.

## Conclusions

In summary, our study did not find a reduction in 28-day in-hospital all-cause mortality in a large cohort of hospitalized patients treated with CCP compared with those untreated with CCP in a multi-center hospital system in Texas. The results should be interpreted in the context of both strengths and limitations of this study, which, while reflecting real-word use of CCP, include a heterogeneous hospitalized patient population largely requiring oxygen support, a lack of data on the antibody titer in the transfused CCP and on the serostatus of patients prior to transfusion. These results can help prioritize future studies and inform the potential role of CP in future pandemics, or in the context of emerging variants, where the rapid availability of CCP, some with ability to cross-neutralize variants [47], supports the importance of continuing to evaluate data from both randomized and observational studies to guide future studies and clinical practices, and optimize patient selection and plasma qualification for CP therapy.

## Supporting information

S1 Appendix Definitions. (DOCX)

S2 Appendix Tables and figures. (DOCX)
