# Supplementary material for: Multi-center matched cohort study of convalescent plasma for hospitalized patients with COVID-19
Source: PLoS One. 2022 Aug 18;17(8):e0273223. doi: 10.1371/journal.pone.0273223 (PMC9387784; doi:10.1371/journal.pone.0273223)
Supplement: S1 Appendix — (DOCX) [file pone.0273223.s001.docx]

**S1 APPENDIX.**

**Table 1. Oxygen requirement strata**

| **Oxygen Requirement Strata** | **Definition** |
| --- | --- |
| Room air | No documentation of the other categories listed below |
| Basic oxygenation | Includes: Simple face mask, nasal cannula, CPAP/Bubble CPAP, T-piece, blow by, nasal prongs, open oxygen mask, tracheostomy collar, venturi mask system (if FiO2 % is < 45%), face tent (if FiO2 % is < 45%; up to 6L), nasal cannula with reservoir (i.e. oximizer; if FiO2 % is < 45%; up to 6L) |
| Advanced oxygenation | Includes: High flow nasal cannula (HFNC), BiPAP/NPPV/NIV, Vapotherm, blendersystem, high flow mask, manual resuscitator, non-rebreather mask (NRB), Oxyhood, partial rebreather mask, venturi mask system (if FiO2 % is > 45%), face tent (if FiO2 % is > 45%; > 7L), nasal cannula with reservoir (i.e. oximizer; if FiO2 % is > 45%; > 7L) |
| Invasive ventilation | Transtracheal catheter, Invasive mechanical ventilation vent mode - CPT code 94002 (mechanical ventilation for initial day), 94003 (subsequent day), 94004 (per day) |
| ECMO | Extracorporeal Membrane Oxygenation |
| NOTE: In cases where clinical flow sheets did not contain sufficient information to classify the level of oxygen support, categorization was based on clinician review of the electronic health record | |

**Table 2. International Society of Blood Transfusion (ISBT)-128 codes for convalescent plasma**

- E9743 Apheresis CONVALESCENT PLASMA|NS/XX/<=-25C|COVID-19
- E9744 Apheresis CONVALESCENT PLASMA|NS/XX/<=-25C|Methylene blue-treated|COVID-19
- E9745 Apheresis CONVALESCENT PLASMA|NS/XX/<=-25C|Psoralen-treated|COVID-19
- E9746 Apheresis CONVALESCENT PLASMA|NS/XX/<=-25C|Riboflavin-treated|COVID-19
- E9747 Apheresis CONVALESCENT PLASMA|ACD-A/XX/<=-18C|COVID-19
- E9748 Apheresis CONVALESCENT PLASMA|NS/XX/Frozen|COVID-19
- E9749 CONVALESCENT PLASMA|NS/XX/Frozen|COVID-19
- E9750 Liquid Apheresis CONVALESCENT PLASMA|NS/XX/refg|COVID-19
- E9751 Liquid CONVALESCENT PLASMA|NS/XX/refg|COVID-19
- E9752 Thawed Apheresis CONVALESCENT PLASMA|ACD-A/XX/refg|COVID-19
- E9764 Thawed Apheresis CONVALESCENT PLASMA|ACD-A/XX/refg|3rd container|COVID-19
- E9763 Thawed Apheresis CONVALESCENT PLASMA|ACD-A/XX/refg|2nd container|COVID-19
- E9754 Apheresis CONVALESCENT PLASMA|ACD-A/XX/<=-18C|1st container|COVID-19
- E9762 Thawed Apheresis CONVALESCENT PLASMA|ACD-A/XX/refg|1st container|COVID-19
- E9765 Thawed Apheresis CONVALESCENT PLASMA|ACD-A/XX/refg|4th container|COVID-19
