# Supplementary material for: Multi-center matched cohort study of convalescent plasma for hospitalized patients with COVID-19
Source: PLoS One. 2022 Aug 18;17(8):e0273223. doi: 10.1371/journal.pone.0273223 (PMC9387784; doi:10.1371/journal.pone.0273223)

**S2 APPENDIX. Tables and Figures**

**S2 Appendix Table 1. ICD-10-CM diagnosis codes to identify safety outcomes**

| **Safety outcomes** | **ICD-10-CM** | **ICD-10-CM Description** |
| --- | --- | --- |
| **Hemovigilance surveillance outcomes** | | |
| Anaphylactic reaction | T80.51XA | Anaphylactic reaction due to administration of blood and blood products, initial encounter |
| Febrile non-hemolytic transfusion reaction | R50.84 | Febrile nonhemolytic transfusion reaction |
| Transfusion-associated circulatory overload (TACO) | E87.71 | TACO |
| Transfusion-related acute lung injury (TRALI) | J95.84 | TRALI |
| Transfusion-transmitted infection | T80.22XA | Acute infection following transfusion, infusion, or injection of blood and blood products, initial encounter |
|  | T80.29XA | Infection following other infusion, transfusion and therapeutic injection, initial encounter |
| Acute hemolytic transfusion reaction (AHTR) | T80.910A | AHTR, unspecified incompatibility, initial encounter |
|  | T80.A10A | Non-ABO incompatibility with AHTR, initial encounter |
| **Cardiac arrhythmias** | | |
| Ventricular tachycardia ventricular arrhythmia | I47.1 | Supraventricular tachycardia |
|  | I47.9 | Ventricular tachycardia |
|  | I47.2 | Paroxysmal tachycardia unspecified |
| Ventricular fibrillation ventricular arrhythmia | I49.01 | Ventricular fibrillation |
| Cardiac arrest | I46.2 | Cardiac arrest due to underlying cardiac condition |
|  | I46.8 | Cardiac arrest due to other underlying condition |
|  | I46.9 | Cardiac arrest, cause unspecified |
| Atrial arrhythmia | I48.0 | Paroxysmal atrial fibrillation |
|  | I48.1 | Persistent atrial fibrillation |

**S2 Appendix Table 2.** Baseline demographics and clinical characteristics of convalescent plasma

treated patients and matched untreated patients through risk-set sampling prior to propensity score matching

|  | CCP Treated | Matched CCP Untreated | Absolute Std Diff |
| --- | --- | --- | --- |
|  | N=1786 | N=3552 |  |
| *Demographics* |  |  |  |
| Age on the index date (years) |  |  | 0.041 |
| Mean (SD) | 64.9 (14.4) | 65.5 (14) |  |
| Median (IQR) | 66 (55, 75) | 66 (56, 76) |  |
| Days from hospital admission to the index date |  |  | 0.01 |
| Mean (SD) | 1.7 (1.7) | 1.7 (1.6) |  |
| Median (IQR) | 1 (1, 2) | 1 (1, 2) |  |
| Sex |  |  | 0.021 |
| Female | 732 (41.0%) | 1493 (42.0%) |  |
| Male | 1054 (59.0%) | 2059 (58.0%) |  |
| Race/ethnicity |  |  | 0.168 |
| Non-Hispanic, White | 959 (53.7%) | 1671 (47.0%) |  |
| Non-Hispanic, Black | 250 (14.0%) | 636 (17.9%) |  |
| Hispanic | 478 (26.8%) | 1089 (30.7%) |  |
| Non-Hispanic, Others | 73 (4.1%) | 103 (2.9%) |  |
| Missing/Unknown | 26 (1.5%) | 53 (1.5%) |  |
| Hospital Category* |  |  | 0.739 |
| >20 ICU beds | 967 (54.1%) | 2590 (72.9%) |  |
| 16-20 ICU beds | 116 (6.5%) | 566 (15.9%) |  |
| 11-15 ICU beds | 455 (25.5%) | 210 (5.9%) |  |
| ≤10 ICU beds | 248 (13.9%) | 186 (5.2%) |  |
| *Comorbidities* |  |  |  |
| History of cancer excluding non-melanoma skin cancer | 123 (6.9%) | 248 (7.0%) | 0.004 |
| Cardiovascular conditions |  |  |  |
| Thrombotic or thromboembolic complications | 311 (17.4%) | 635 (17.9%) | 0.012 |
| Stroke | 169 (9.5%) | 394 (11.1%) | 0.054 |
| Myocardial infarction | 111 (6.2%) | 222 (6.3%) | 0.001 |
| Venous thromboembolism, Deep vein thrombosis, Pulmonary embolism | 74 (4.1%) | 142 (4.0%) | 0.007 |
| Hypertension | 1236 (69.2%) | 2536 (71.4%) | 0.048 |
| Heart failure | 364 (20.4%) | 810 (22.8%) | 0.059 |
| Cardiac arrhythmias | 340 (19.0%) | 651 (18.3%) | 0.018 |
| Chronic respiratory disease |  |  |  |
| Chronic obstructive pulmonary disease | 316 (17.7%) | 642 (18.1%) | 0.01 |
| Asthma | 204 (11.4%) | 334 (9.4%) | 0.066 |
| Diabetes mellitus | 927 (51.9%) | 1911 (53.8%) | 0.038 |
| Chronic kidney disease |  |  | 0.092 |
| 1 to 4 | 403 (22.6%) | 749 (21.1%) |  |
| 5 or end-stage renal disease | 92 (5.2%) | 232 (6.5%) |  |
| Chronic liver disease | 132 (7.4%) | 239 (6.7%) | 0.026 |
| History of organ transplantation | 53 (3.0%) | 65 (1.8%) | 0.074 |
| HIV/AIDS | 2 (0.1%) | 12 (0.3%) | 0.048 |
| Obesity at hospital admission |  |  | 0.187 |
| BMI 30-39.9 | 756 (42.3%) | 1294 (36.4%) |  |
| BMI ≥40 | 248 (13.9%) | 494 (13.9%) |  |
| Missing | 3 (0.2%) | 17 (0.5%) |  |
| Sickle cell disease† | 1 (0.1%) | 4 (0.1%) | 0.02 |
| Pregnant from index date to study completion | 7 (0.4%) | 20 (0.6%) | 0.025 |
| *Comedications* |  |  |  |
| Antiviral drugs |  |  |  |
| Remdesivir | 1268 (71.0%) | 1952 (55.0%) | 0.337 |
| Lopinavir/Ritonavir † | 1 (0.1%) | 0 (0%) | 0.034 |
| Other HIV protease inhibitors † | 4 (0.2%) | 20 (0.6%) | 0.054 |
| Hydroxychloroquine/Chloroquine | 12 (0.7%) | 24 (0.7%) | 0.001 |
| Azithromycin | 1007 (56.4%) | 1723 (48.5%) | 0.158 |
| Glucocorticoid/Steroids |  |  |  |
| Dexamethasone | 1716 (96.1%) | 3429 (96.5%) | 0.024 |
| Prednisone | 126 (7.1%) | 285 (8.0%) | 0.037 |
| Hydrocortisone | 70 (3.9%) | 145 (4.1%) | 0.008 |
| Anti-platelet agents | 701 (39.2%) | 1381 (38.9%) | 0.008 |
| IL-6 inhibitor/antagonist |  |  |  |
| Tocilizumab | 111 (6.2%) | 110 (3.1%) | 0.148 |
| Sarilumab † | 0 (0%) | 0 (0%) |  |
| Siltuximab † | 0 (0%) | 0 (0%) |  |
| ACE-Inhibitors/Angiotensin Receptor Blockers | 527 (29.5%) | 1036 (29.2%) | 0.008 |
| Anti-thrombotic drugs | 1624 (90.9%) | 3099 (87.2%) | 0.118 |
| *Clinical Characteristics* |  |  |  |
| Oxygenation support 4-12 hours prior to the index date |  |  | 0.141 |
| Room air | 204 (11.4%) | 397 (11.2%) |  |
| Basic oxygen support | 1029 (57.6%) | 2271 (63.9%) |  |
| Advanced oxygen support | 503 (28.2%) | 821 (23.1%) |  |
| Invasive ventilation | 50 (2.8%) | 63 (1.8%) |  |
| ECMO | 0 (0%) | 0 (0%) |  |
| ICU admission prior to index date | 211 (11.1%) | 494 (11.9%) | 0.054 |
| Vital signs closest to the index date |  |  |  |
| Respiratory rates |  |  | 0.092 |
| Mean (SD) | 21.0 (5.6) | 20.4 (5.8) |  |
| Median (IQR) | 20 (18, 22) | 20 (18, 22) |  |
| Heart rate |  |  | 0.001 |
| Mean (SD) | 78.5 (15.6) | 78.5 (16.2) |  |
| Median (IQR) | 77 (67, 88) | 77 (67, 88) |  |
| Systolic blood pressure |  |  | 0.056 |
| Mean (SD) | 130.0 (20.2) | 128.8 (19.9) |  |
| Median (IQR) | 128 (116, 144) | 127 (115, 142) |  |
| Temperature (F) |  |  | 0.004 |
| Mean (SD) | 98.3 (0.8) | 98.3 (0.8) |  |
| Median (IQR) | 98.2 (97.8, 98.6) | 98.2 (97.8, 98.6) |  |
| Laboratory results closest to the index date |  |  |  |
| Creatinine Level (mg/dL) |  |  | 0.063 |
| ≤ 1.5 | 1413 (79.1%) | 2734 (77.0%) |  |
| >1.5 | 320 (17.9%) | 669 (18.8%) |  |
| Missing | 53 (3%) | 149 (4.2%) |  |
| D-dimer Level (ug/mL FEU) |  |  | 0.086 |
| ≤ 1 | 806 (45.1%) | 1519 (42.8%) |  |
| 1 - 2 | 376 (21.1%) | 686 (19.3%) |  |
| >2 | 272 (15.2%) | 593 (16.7%) |  |
| Missing | 332 (18.6%) | 754 (21.2%) |  |
| Cardiac troponin Level - Tnl (98%) or TnT (2%) (ng/mL) |  |  | 0.088 |
| ≤ 1 | 1249 (69.9%) | 2325 (65.5%) |  |
| > 1 | 36 (2.0%) | 73 (2.1%) |  |
| Missing | 501 (28.1%) | 1154 (32.5%) |  |
| Absolute lymphocyte count Level (K/uL) |  |  | 0.164 |
| <1 | 445 (67.5%) | 1017 (53.6%) |  |
| ≥1 | 505 (28.3%) | 1185 (33.4%) |  |
| Missing | 98 (5.5%) | 282 (7.9%) |  |
| Ferritin Level (ng/mL) |  |  | 0.094 |
| ≤400 | 403 (22.6%) | 901 (25.4%) |  |
| >400 | 890 (49.8%) | 1683 (47.4%) |  |
| Missing | 493 (27.6%) | 968 (27.3%) |  |
| C-reactive protein Level (mg/dL) |  |  | 0.024 |
| ≤0.5 | 28 (1.6%) | 76 (2.1%) |  |
| >0.5 | 1358 (76.0%) | 2739 (77.1%) |  |
| Missing | 400 (22.4%) | 737 (20.7%) |  |

Abbreviation: ACE = angiotensin converting enzyme; BMI = body mass index; CCP = COVID-19 convalescent plasma; ECMO = extracorporeal membrane oxygenation; FEU = Forty-foot equivalent unit; ICU = intensive care unit; IQR = interquartile range; SD = standard deviation; Std diff = Standardized difference; Tn = Cardiac troponin.

*Hospital category was not included in the propensity score model but included as a random effect in the outcome regression.

† Characteristics were not included in the propensity score model due to insufficient sample size.

**S2 Appendix Table 3. Safety events related to plasma transfusion at day 28 post the index date in the post-propensity score matching cohort**

|  | CCP Treated | Matched CCP Untreated |
| --- | --- | --- |
|  | **N=1245** | **N=1245** |
|  | **n (%)** | **n (%)** |
| Transfusion associated circulatory overload | 1 (0.1%) | 0 (0%) |
| Transfusion-related acute lung injury | 0 (0%) | 0 (0%) |
| Anaphylactic reaction | 0 (0%) | 0 (0%) |
| Febrile non-hemolytic transfusion reaction | 0 (0%) | 0 (0%) |
| Transfusion-transmitted infection | 0 (0%) | 0 (0%) |
| Acute hemolytic transfusion reaction | 0 (0%) | 0 (0%) |
| Thrombotic or thromboembolic complication* | 140 (11.2%) | 131 (10.5%) |
| Cardiac arrhythmias ^†^ | 161 (12.9%) | 171 (13.7%) |

Abbreviations: CCP = COVID-19 convalescent plasma

* Thrombotic or thromboembolic complication includes stroke, myocardial infarction, venous thromboembolism, deep vein thrombosis, and pulmonary embolism

^†^ Cardiac arrhythmias include ventricular tachycardia, ventricular fibrillation, atrial arrhythmia, and cardiac arrest

**S2 Appendix Table 4.** Incidence rates of in-hospital mortality in the pre- and post-propensity score matched cohorts

| In-hospital deaths from the index date | CCP Treated | | | | |  | Matched CCP Untreated | | | | |
| --- | --- | --- | --- | --- | --- | --- | --- | --- | --- | --- | --- |
|  | **Sample size** | **No. of events** | **Person-time** | **IR** | **95% CI** |  | **Sample size** | **No. of events** | **Person-time** | **IR** | **95% CI** |
| *Pre-propensity score matched cohort** | 1786 |  |  |  |  |  | 3552 |  |  |  |  |
| Day 7 |  | 86 | 9126 | 9.4 | (7.6, 11.6) |  |  | 132 | 16193 | 8.2 | (6.9, 9.7) |
| Day 14 |  | 182 | 12713 | 14.3 | (12.4, 16.5) |  |  | 270 | 21515 | 12.5 | (11.1, 14.1) |
| Day 21 |  | 264 | 14278 | 18.5 | (16.4, 20.8) |  |  | 348 | 23981 | 14.5 | (13.1, 16.1) |
| Day 28 |  | 304 | 14961 | 20.3 | (18.2, 22.7) |  |  | 400 | 25316 | 15.8 | (14.3, 17.4) |
| *Post-propensity score matched cohort* | 1245 |  |  |  |  |  | 1245 |  |  |  |  |
| Day 7 |  | 51 | 6211 | 8.2 | (6.2, 10.8) |  |  | 49 | 5935 | 8.3 | (6.2, 10.9) |
| Day 14 |  | 110 | 8523 | 12.9 | (10.7, 15.5) |  |  | 96 | 7827 | 12.3 | (10.1, 15.0) |
| Day 21 |  | 156 | 9484 | 16.4 | (14.1, 19.2) |  |  | 127 | 8681 | 14.6 | (12.3, 17.4) |
| Day 28 |  | 179 | 9905 | 18.1 | (15.6, 20.9) |  |  | 143 | 9109 | 15.7 | (13.3, 18.5) |

Abbreviations: CCP = COVID-19 convalescent plasma; IR = incidence rate per 1000 person days; CI = confidence interval

* The cohort after risk-set sampling and before propensity score matching.

**S2 Appendix Table 5.** Discharge disposition for patients discharged alive within 28 days post index date

| Discharge Disposition | CCP Treated | Matched CCP Untreated |
| --- | --- | --- |
|  | **N=1029** | **N=1063** |
|  | **n (%)** | **n (%)** |
| Home or Self Care | 664 (64.6%) | 724 (68.1%) |
| Home with Home Health | 159 (15.5%) | 154 (14.5%) |
| Skilled Nursing Facility | 103 (10%) | 105 (9.9%) |
| Long Term Acute Care | 38 (3.7%) | 20 (1.9%) |
| Rehabilitation Facility | 25 (2.4%) | 25 (2.4%) |
| Home with Hospice | 8 (0.8%) | 10 (0.9%) |
| Inpatient Hospice | 8 (0.8%) | 10 (0.9%) |
| Short Term Hospital | 10 (1%) | 3 (0.3%) |
| Nursing Facility | 4 (0.4%) | 5 (0.5%) |
| Court/Law Enforcement | 4 (0.4%) | 2 (0.2%) |
| Left Against Medical Advice/Discontinued Care/Eloped | 3 (0.3%) | 4 (0.4%) |
| Discharged to another facility | 2 (0.2%) | 1 (0.1%) |
| Psychiatric Hospital | 1 (0.1%) | 0 (0%) |

Abbreviations: CCP = COVID-19 convalescent plasma

**S2 Appendix Figure 1. Density plot of propensity scores in patients treated with COVID-19 convalescent plasma (CCP) and those not treated pre- and post-propensity score matching**


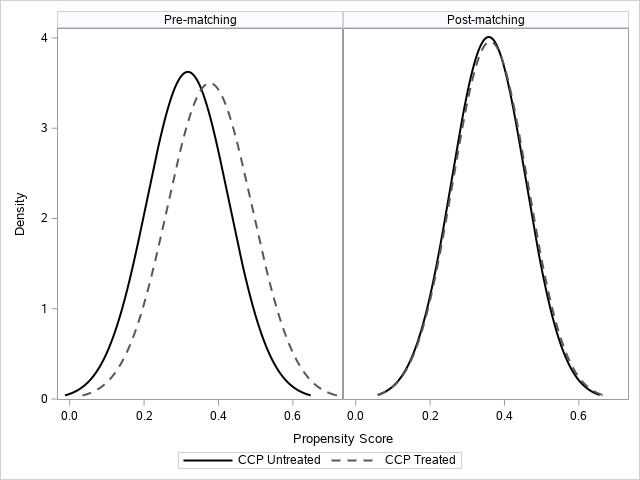


**S2 Appendix Figure 2. Distribution of COVID-19 convalescent plasma (CCP) transfusion by calendar month**

February 4, 2021: EUA restricted to high antibody titer plasma

August 23, 2020: CCP authorized under Emergency Use Authorization (EUA)

COVID-19 convalescent plasma (CCP) was authorized by US FDA under Emergency Use Authorization (EUA) on August 23, 2020 for the treatment of COVID-19 in hospitalized patients (38). On February 4, 2021, based on updated findings of randomized controlled trials (RCTs), the EUA for CCP was updated to restrict the use of CCP to high antibody titer plasma used early in the course of disease or in patients with impaired humoral immunity (39).

**S2 Appendix Figure 3. Cumulative in-hospital mortality up to 28 days after index date with censoring discharge alive at day 28**


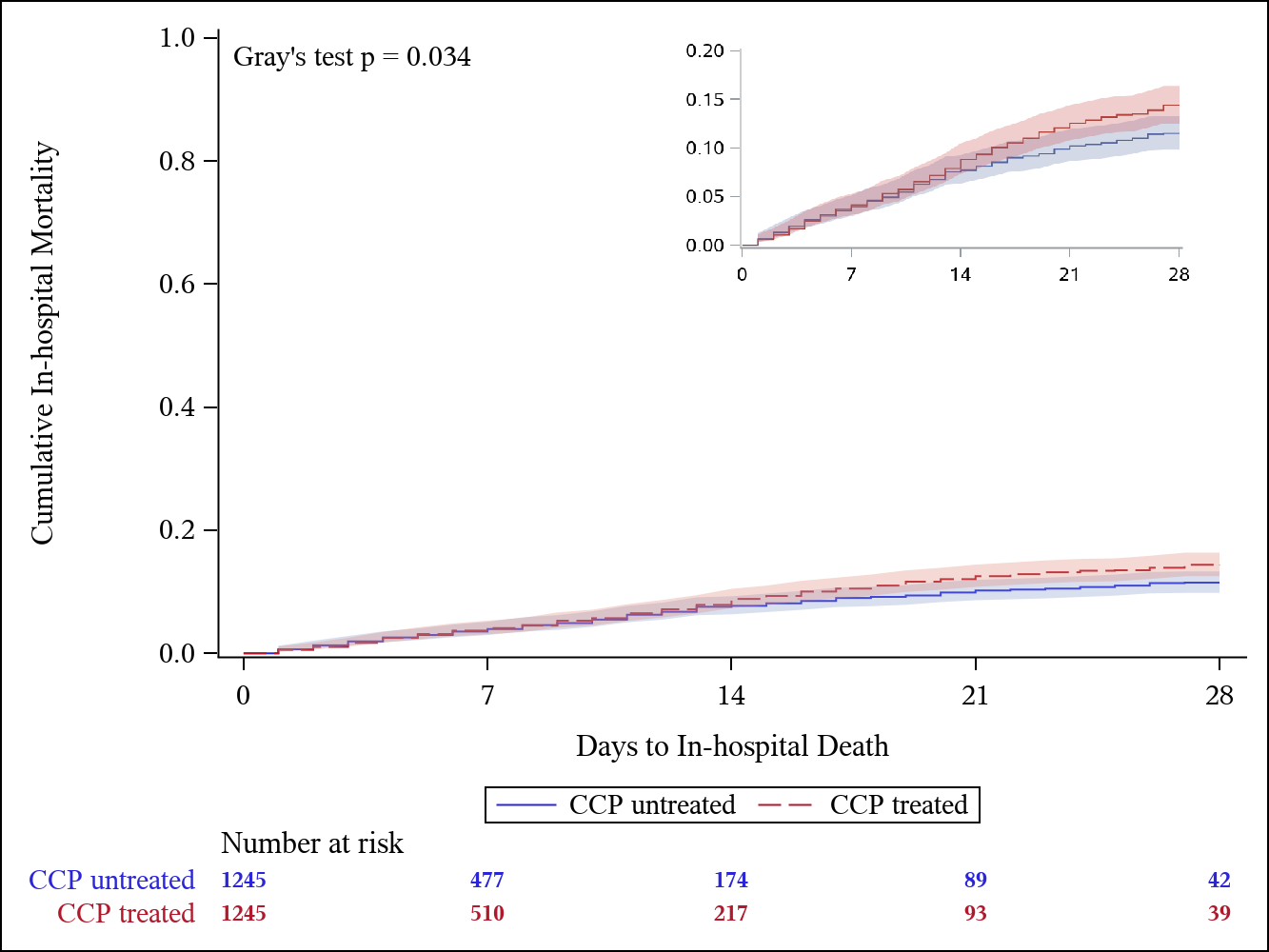

Supplement: S2 Appendix — (DOCX) [file pone.0273223.s002.docx]
